# Genomic Signatures of Reinforcement

**DOI:** 10.3390/genes9040191

**Published:** 2018-04-03

**Authors:** Austin G. Garner, Benjamin E. Goulet, Matthew C. Farnitano, Y. Franchesco Molina-Henao, Robin Hopkins

**Affiliations:** 1 Department of Organismic and Evolutionary Biology, Harvard University, Cambridge, MA 021382, USA; aggarner@g.harvard.edu (A.G.G.); bgoulet@g.harvard.edu (B.E.G.); mfarnitano@fas.harvard.edu (M.C.F.); molinahenao@fas.harvard.edu (Y.F.M.-H.); 2 The Arnold Arboretum, Harvard University, Boston, MA 02131, USA; 3 Department of Biology, Universidad del Valle, Cali 760032, Colombia

**Keywords:** reinforcement, speciation, selection, genetic divergence, gene flow, reproductive isolation, genome scans

## Abstract

Reinforcement is the process by which selection against hybridization increases reproductive isolation between taxa. Much research has focused on demonstrating the existence of reinforcement, yet relatively little is known about the genetic basis of reinforcement or the evolutionary conditions under which reinforcement can occur. Inspired by reinforcement’s characteristic phenotypic pattern of reproductive trait divergence in sympatry but not in allopatry, we discuss whether reinforcement also leaves a distinct genomic pattern. First, we describe three patterns of genetic variation we expect as a consequence of reinforcement. Then, we discuss a set of alternative processes and complicating factors that may make the identification of reinforcement at the genomic level difficult. Finally, we consider how genomic analyses can be leveraged to inform if and to what extent reinforcement evolved in the face of gene flow between sympatric lineages and between allopatric and sympatric populations of the same lineage. Our major goals are to understand if genome scans for particular patterns of genetic variation could identify reinforcement, isolate the genetic basis of reinforcement, or infer the conditions under which reinforcement evolved.

## 1. Introduction

The evolution of barriers to reproduction between lineages is fundamental to the process of speciation. Recent advancements in genome sequencing technologies and analyses have improved our ability to identify and characterize the genetic and evolutionary mechanisms underlying these reproductive isolating barriers. Genomic studies have extensively focused on understanding how barriers evolved in response to natural selection for local adaptation (e.g., [1,2,3,4]); however, selection also plays an important role in speciation through the process of reinforcement. Here we outline the genomic patterns of variation that may result from reinforcement and discuss what we can and cannot learn about the evolutionary processes underlying reinforcement from genomic variation.

Reinforcement is the process by which reproductive isolation increases between taxa in sympatry due to natural selection against hybridization [5]. A variety of definitions of reinforcement have been used [5,6,7,8], but here we use a broad definition that encompasses the evolution of traits that decrease hybridization or the production of hybrids between closely related lineages due to selection against unfit hybrid progeny or costly mating. Since Dobzhansky’s instrumental work on the topic [9,10,11] evidence for reinforcement has accumulated from across the tree of life, including in plants [12,13,14], fish [15,16,17], insects [18,19,20,21,22], amphibians [23,24,25,26], birds [27], and mammals [28,29]. In all of these examples, divergence in at least one species causes increased reproductive isolation in sympatric populations of a closely related species. Although there are other scenarios by which reinforcement can occur (e.g., the one-allele model; see Section 3.3), most of our discussion centers on a simplified evolutionary scenario consistent with what is predominantly studied in nature. The number of examples of reinforcement observed across the tree of life suggests the potential importance of reinforcement for the formation and stability of species, yet there remains much to be learned about the prevalence of reinforcement, the genetic basis of traits involved, and the strength of selection and gene flow during reinforcement’s evolution [6,7,30,31,32,33].

Investigating genomic patterns associated with reinforcement may provide new insights into when and how reinforcement evolves. Identifying targets of selection in the genome and inferring evolutionary processes acting on genetic loci is becoming increasingly feasible through advancements in sequencing technology and analytical methods [34,35,36,37]. These genomic approaches have revealed the genetic basis and evolutionary history of traits conferring reproductive isolation [38,39]. We hypothesize the evolution of reinforcement can leave an identifiable genomic signature. Therefore, genomic analyses may help to identify the process of reinforcement in natural populations and isolate the genetic loci underlying reinforcement traits. This approach might be especially useful when the reinforcement phenotype is cryptic or not easily studied in the lab. Some efforts have been made to identify the genetic basis of reinforcement [13,40,41,42,43], and a few studies have searched for signatures of selection at known [44] and putative [43,45] reinforcement loci. These studies motivate the need to articulate the expected genomic signature of reinforcement and discuss the potential insights we can gain from genomic studies of this process.

First, we describe the expected genomic and population genetic signatures of reinforcement. Second, we discuss alternative causes of these genomic patterns and how these alternatives might be differentiated from reinforcement. Third, we explore a myriad of factors that can interact with reinforcement’s genomic signature, making it easier or more difficult to identify. Finally, we address how genomic analyses of gene flow can be used to understand how reinforcement evolves.

## 2. The Genomic Signature of Reinforcement

**Figure 1:**
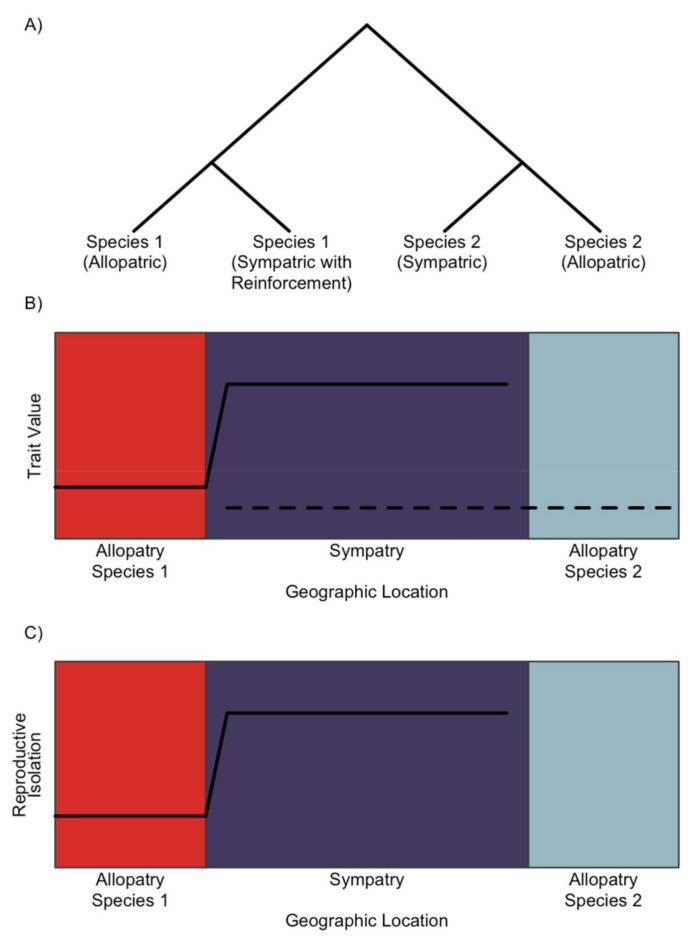
The phenotypic pattern of reinforcement. (**a**) A schematic of the relationship between two species each with sympatric and allopatric populations. Secondary contact between the lineages in sympatry results in costly hybridization, causing reinforcement in Species 1. (**b**) Reinforcement selects for a new reproductive trait value in Species 1 (solid line) to prevent mating with Species 2 (dashed line) in sympatry (purple). Allopatric populations of Species 1 (red) retain the ancestral phenotype. In this simplified scenario, Species 2 does not change trait value in sympatry or allopatry (blue), but in some cases Species 2 may also diverge in trait value in sympatry. (**c**) For Species 1, reinforcement increases reproductive isolation (solid line) with Species 2 in sympatry relative to allopatry.

Reinforcement often results in a pattern of character displacement, with greater reproductive isolation in sympatric populations compared to allopatric populations (Figure 1) [5,6,7,32]. This well-studied phenotypic pattern motivates us to search for a similarly distinctive genomic pattern associated with reinforcement. The process of reinforcement can be described as a simplified scenario that starts with two lineages diverging in allopatry or with limited gene flow. After accumulating some postzygotic barriers to reproduction resulting from intrinsic or extrinsic incompatibilities, these two lineages come into secondary contact in a region of sympatry. Prezygotic reproductive isolation is not complete, allowing for costly hybridization between the diverged lineages. The reduction in fitness due to hybridization generates indirect selection favoring traits that increase prezygotic reproductive isolation. A novel trait value that reduces hybridization can spread throughout sympatry and create the pattern of greater reproductive isolation in sympatry than allopatry (Figure 1) [46,47,48,49]. For this discussion, we will often refer to these diverging lineages as ‘species’. This taxonomic distinction is arbitrary and used for simplicity.

What might this classic scenario of reinforcement look like at the genetic level? Two isolated lineages will accumulate genetic divergence through drift and natural selection. Some of these genetic differences will result in intrinsic or extrinsic incompatibilities between lineages. Upon secondary contact, reinforcing selection will favor mutations within a lineage that decrease maladaptive hybridization between the two lineages. This selection will drive the mutations associated with increased prezygotic reproductive isolation to fixation or high frequency throughout the sympatric portion of a lineage’s range.

**Figure 2:**
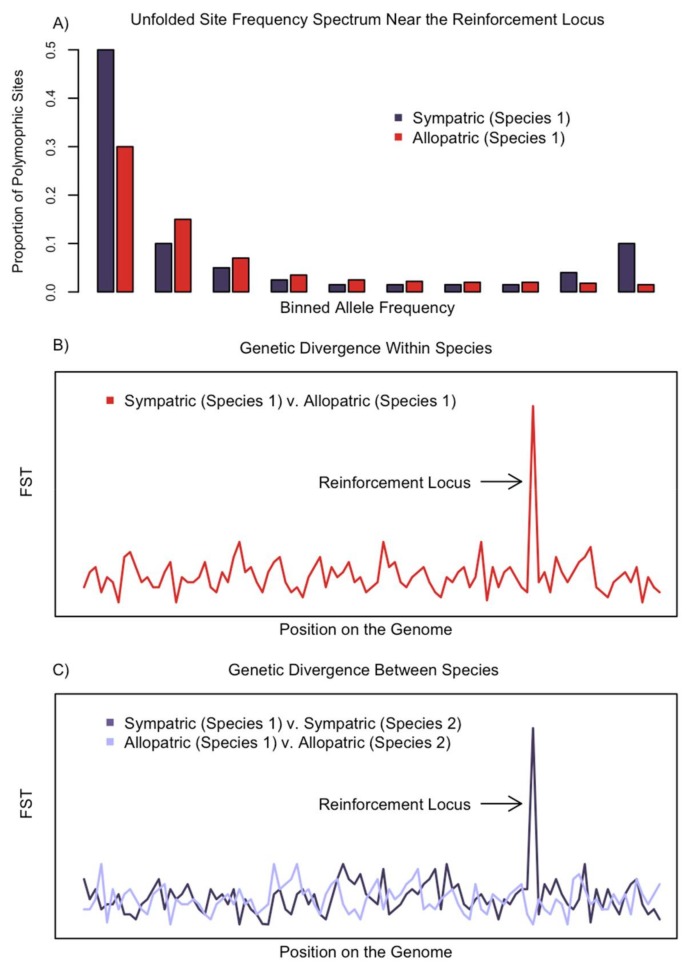
Hypothetical examples of three genomic signatures that may result from the evolution of reinforcement. (**a**) Reinforcement can cause a signature of a selective sweep in sympatry, distorting the site frequency spectrum near the reinforcement locus. In sympatric populations, there will be an excess of fixed and rare alleles compared to allopatric populations. (**b**) Trait divergence in sympatric populations can cause increased genetic divergence around the reinforcement locus between allopatric and sympatric populations of the same species. Both an increase in absolute divergence between allopatric and sympatric populations and a decrease in diversity within sympatric populations can cause elevated F_ST_ near reinforcement loci. (**c**) Reinforcement only selects for divergence between hybridizing sympatric species and not their allopatric counterparts. This pattern can cause greater divergence (F_ST_) between sympatric populations of the two species compared to allopatric populations of the two species at the reinforcement locus. Increased F_ST_ could be caused by both greater divergence between sympatric alleles and less allelic diversity within a sympatric population.

From this simplified genetic scenario of reinforcement emerge three predictions about the patterns of genetic variation within and between two sympatric species (Figure 2). (1) In the species that undergoes trait divergence due to reinforcement, we expect a genomic signature of selection surrounding mutations that cause increased reproductive isolation in sympatric individuals, but no signature in allopatric individuals. (2) We predict elevated genetic divergence between allopatric and sympatric individuals of the same species at loci causing increased prezygotic reproductive isolation. (3) We expect greater genetic divergence between the sympatric than the allopatric individuals of different species at genomic regions causing increased prezygotic reproductive isolation. These three predicted patterns of genetic variation are related as they arise from the same process, selection favoring divergence in sympatry.

A rapid increase in frequency of a reinforcement allele due to selection is expected to distort variation in linked genetic sites causing a genomic signature of a selective sweep [34,50,51]. This signature will be strongest at the site of selection and dissipate with distance. A selective sweep is not dependent on the source of selection and therefore we will not review how to detect a signature of selection as that has been done elsewhere [37,52,53,54]. In general, we expect a signature of selection characterized by reduced genetic variation [55], increased linkage disequilibrium or haplotype blocks [56,57,58,59], and a skewed site frequency spectrum [34,60,61] surrounding the causal mutations (Figure 2a). These patterns of genetic variation can be summarized using statistics such as Tajima’s D [62], Fay and Wu’s H [60], ω-statistic [57], and integrated haplotype score (iHS) [63] that are calculated and analyzed across the genome using programs such as SweepFinder2 [64], SweepD [65], and OmegaPlus [66]. The predicted signature may vary depending on the evolutionary history of the alleles under selection (see Section 4.1 for a discussion on variation between soft and hard sweeps). Importantly, under our hypothetical scenario, we expect these patterns of selection to only occur in sympatry and not allopatry.

Traits favored by reinforcement in sympatry are often costly or not favored in allopatry [67,68], leading to the phenotypic pattern of character displacement. As with any trait under divergent selection across geographic space, we predict elevated genetic divergence between alleles at the reinforcement loci in allopatric and sympatric populations. Scanning the genome for outliers of summary statistics such as F_ST_ [69] and derivatives of F_ST_ [70] can reveal candidate loci under divergent selection. F_ST_ is a relative measure of divergence and therefore an increase in this summary statistic can be caused by both increased allelic divergence between allopatric and sympatric populations and also a decrease in allelic diversity within populations [71].

Character displacement, whether due to reinforcement or competition is defined by greater phenotypic divergence between two species in sympatry relative to divergence in allopatry [47,72]. The same pattern of divergence may be evident at the genomic level as well. During character displacement, derived alleles cause phenotypic divergence in sympatric populations, implying that allopatric individuals from both species retain ancestral alleles or alleles that have undergone less genetic divergence since the most recent common ancestor of the two species. Therefore, sympatric individuals of different species may have greater measures of genetic divergence (F_ST_) than allopatric individuals of these species at reinforcement loci. As above, elevated F_ST_ may result from increased absolute divergence between species at alleles in sympatry or due to decreased allelic diversity within one or both species in sympatric populations. Significant absolute allelic divergence may be hard to detect depending on the number of mutations in a particular allele, the average divergence between species, and the allelic diversity within each species. Therefore, if this pattern is not observed, it does not mean reinforcement did not evolve.

We know of only two systems for which a genetic signature of reinforcement has been investigated [44,45]. In *Phlox*, *P. cuspidata* and *P. drummondii* exhibit similar light-blue flowers throughout their allopatric ranges, but when these species occur in sympatry, *P. drummondii* has evolved dark-red flowers to prevent hybridization with *P. cuspidata* [73]. The flower color divergence in *P. drummondii* is caused by cis-regulatory changes to two genes in the anthocyanin biosynthesis pathway, flavanone-3′5′-hydroxylase (*F3′5′h*) and *R2R3-Myb* transcription factor [13]. Genetic diversity (π) in the coding sequence of *F3′5′h* within sympatric populations is less than within allopatric populations suggesting a possible history of selection at this locus [44]. All other genetic tests for selection suggest that these two flower color genes evolved neutrally. The mutations causing expression variation in these flower color genes are still unknown leaving open the possibility that genetic variation surrounding the causal sites may support a history of selection. The second study by Smadja et al. [45] investigates plausible candidate genes involved in assortative mating in the hybrid zone between *Mus musculus musculus* and *M. m. domesticus*. They use the lnRH statistic [74,75] to evaluate allele frequency variation in microsatellites in or near genes involved in pheromone signaling and olfactory recognition, and they find a number of regions showing significant patterns of reduced variability in a *M. m. musculus* ‘contact’ population, adjacent to the hybrid zone, that has elevated assortative mating relative to an allopatric population of *M. m. musculus*. These patterns of variation suggest candidate genes experienced recent selection in populations near the zone of contact but not in allopatric populations. While both of these studies have laid the foundation for identifying and characterizing reinforcement at the genomic level, to date this field remains largely unexplored.

## 3. Alternative Hypotheses to the Signatures of Reinforcement

Linking genomic patterns of diversity and divergence to their causal evolutionary processes remains an ongoing challenge. Although we have described several expectations for a genomic signature of reinforcement, other processes can create a similar phenotypic pattern of character displacement [5,30,46,72,76], and therefore may leave similar genomic patterns of variation. Here, we discuss factors that may mimic the patterns of genetic divergence associated with reinforcement. With appropriate experimental design and genomic analysis, reinforcement may be differentiated from some of these factors. However, reinforcement may evolve as a secondary result of or in tandem with some of these processes, and therefore reinforcement may not be separable from other evolutionary mechanisms [32].

### 3.1. Ecological Character Displacement

Ecological character displacement (ECD) is the process of divergence between sympatric taxa due to selection to reduce competition (e.g., for food, space, shelter) [47,72,77,78,79]. Phenotypically, this process can mimic reinforcement. ECD occurs when two divergent lineages come into secondary contact and occupy overlapping ecological niches. Selection to decrease resource competition favors trait divergence. This process results in a phenotypic pattern of increased trait divergence between sympatric populations, where there is competition, but not in allopatric populations, where competition is absent. The resulting trait divergence can involve traits associated with mate choice or assortative mating, such as mating calls, flowering time, habitat choice, that may or may not cause prezygotic reproductive isolation [30,80]. Therefore, without identifying the source of selection, differentiating reinforcement from ECD can be difficult [15].

ECD and reinforcement can also leave similar patterns of genetic variation across the genome. In sympatry, selection due to interspecific competition increases the frequency of alleles causing genetic divergence, potentially resulting in a selective sweep. Divergent selection between sympatry, where there is competition, and allopatry, where there is not competition, can generate elevated genetic divergence at loci underlying ECD. If sympatric species are closely related and similarities in homologous traits cause niche overlap, then we might expect greater genetic divergence between species at loci causing ECD in sympatry than in allopatry. These three genomic patterns of variation mirror the expected genomic patterns of variation from reinforcement. Therefore, to differentiate between ECD and reinforcement it is necessary to identify the source of selection causing genetic divergence. Without understanding the biology of the interactions all that can be identified is a genetic pattern of character displacement.

### 3.2. Local Adaptation

Patterns of phenotypic divergence, and therefore genetic divergence, due to local adaptation can closely resemble patterns caused by reinforcement. Local adaptation is the process by which a population of individuals evolves higher fitness in a resident environment relative to individuals from other environments [81,82]. Local adaptation is characterized by phenotypic and genetic differentiation along environmental gradients or across diverging habitats [83,84,85]. During the process of local adaptation, natural selection can sweep favored alleles to fixation within populations, generating genomic divergence between populations experiencing different environments. Sympatric and allopatric populations are, by definition, geographically distinct. Selective sweeps in sympatry and genetic divergence between sympatric and allopatric populations within a species may arise due to local adaptation to the sympatric environment rather than selection against costly hybridization with a sympatric species.

Although selection due to local adaptation and selection due to reinforcement can leave similar patterns of genetic variation, it may be possible to disentangle the causal evolutionary force. Candidate loci underlying local adaptation are often identified by correlations between allele frequencies and environmental variables across populations [86,87,88,89,90]. Some environmental gradients may be perfectly aligned with the presence/absence of a closely related species, but in most cases thoughtful sampling can identify populations across which selection due to hybridization and selection due to a dominant environmental variable are not perfectly correlated. If populations spanning variable environments within allopatry and sympatry are sampled, then statistical models could potentially identify loci that are more strongly associated with region (allopatric vs. sympatry) than with environment. Smadja et al. [45] did this to some extent by comparing an allopatric population of *M. m. musculus* near a population close to the contact zone with an allopatric population far from the zone of contact. Loci that showed elevated divergence between these two allopatric populations were eliminated as reinforcement candidates even if they showed high divergence in the ‘contact’ population. Ideally, multiple populations in both allopatry and sympatry that span environmental variation would be used in this type of analysis.

In some cases, environmental variability may be inseparable from the presence of a heterospecific species thus making inferring the mechanism of selection impossible from genomic data. Furthermore, theoretical models have demonstrated that reinforcement is more likely to be successful if loci that decrease hybridization are also under direct selection in the sympatric environment [91,92]. This finding suggests that divergence between sympatry and allopatry (at both the phenotypic and genetic level) could likely be due to both local adaptation and reinforcement. For this reason, experiments that measure selection in natural settings and can therefore identify the source or sources of selection (e.g., [73]) are needed to validate genomic signatures.

### 3.3. Adaptive Introgression and the One-Allele Model

Adaptive introgression occurs when a genetic variant that was introduced into a population via gene flow from a distinct group is favored by natural selection [93]. Demonstrating that adaptive introgression has occurred requires both evidence that a trait is adaptive and that the adaptive allele spread between species. At the genomic level, regions sharing signatures of selection and introgression are compelling candidates for loci involved in adaptive introgression [94,95,96]. Adaptive introgression may be indistinguishable at the genomic level from the one-allele model of reinforcement.

Our simple scenario of reinforcement involves the divergent evolution of a trait value that increases reproductive isolation between sympatric populations of two species. This model is a two-allele model of reinforcement as it assumes that the two sympatric species have different alleles at the loci causing reproductive isolation [97]. By contrast, the one-allele model of reinforcement assumes that a single allele increases reproductive isolation when it occurs in both of the two sympatric taxa [33,97,98]. For example, an allele that increases assortative mating between phenotypically similar individuals could be favored by reinforcing selection in both sympatric species if these sympatric species are phenotypically distinct. Evidence for such a system has been documented in *Drosophila*, in which a single allele confers female choosiness in the genetic background of both sympatric species [99].

In cases of adaptive introgression, sympatric populations of two distinct groups will, by definition, share the same adaptive allele at a locus underlying the adaptive trait. This genetic pattern contrasts with the expected pattern for the two-allele model of reinforcement, in which sympatric populations of distinct groups will have different alleles at a locus involved in reproductive isolation. However, one-allele reinforcement resembles adaptive introgression. In fact, the one-allele model of reinforcement is a special case of adaptive introgression in which the source of selection (costly hybridization) and involved traits (barriers to reproduction) are specified. If an allele arises in one species and spreads into the sympatric population of another species, then it is an example of introgression. If this shared allele is favored by selection in both populations because it decreases costly hybridization, then it is adaptive. Therefore, population genomic data may be used to identify putative examples of the one-allele model of reinforcement by combining methods developed to search for signatures of introgression and selection, as have been applied to searches for adaptive introgression more generally. Notably, the reinforcement allele underlying the one-allele model of reinforcement is only advantageous in the zone of sympatry, so while there will be no genetic divergence between sympatric species at the reinforcement locus, there should still be genetic divergence between the sympatric and allopatric populations of the same species. However, this signature is not exclusive to the one-allele model of reinforcement as loci conferring adaptation to the sympatric environment would show a similar pattern. Genomic data are therefore unlikely to be able to distinguish one-allele reinforcement from other cases of adaptive introgression because the criteria that make the one-allele model a unique case (source of selection and nature of involved traits) are not associated with diagnostic genomic signatures.

## 4. Complicating Factors to Identifying Genomic Signatures of Reinforcement

Patterns of genomic divergence can be variable even in the absence of selective processes. Here we discuss how the history of a mutation and variation in recombination rate could further complicate the identification of reinforcement from genomic data.

### 4.1. History of Mutation

The evolutionary history of a selectively favored allele affects the patterns of genetic diversity surrounding that allele. Adaptive alleles arise either as new mutations or exist as standing genetic variation within a population prior to being a target of selection. These two scenarios tend to give rise to different signatures of selection (i.e., a hard selective sweep or a soft selective sweep respectively) [100]. Here we focus solely on how the evolutionary history of a mutation specifically affects reinforcement.

Reproductive isolation may be caused by a new mutation in sympatry, standing genetic variation prior to colonization in sympatry, or by migration of an allele from allopatry into sympatry. The dynamics of reinforcement may make the evolution from standing genetic variation more plausible. When two species spread into secondary contact hybridization can cause their fusion, extinction of one or both of the species, or reinforcement [46]. Successful reinforcement depends on premating isolation evolving before the taxa fuse or go extinct. Therefore, reinforcement may be more successful if an allele causing increased premating reproductive isolation exists at low frequency prior to reinforcing selection. Under this scenario, the causal mutation under selection may recombine into multiple genetic backgrounds prior to increasing in frequency, resulting in a soft selective sweep.

Soft selective sweeps are more difficult to identify and therefore may go undetected by frequency- and diversity-based methods compared to haplotype-based methods [37,53]. If alleles causing increased reproductive isolation arose as standing genetic variation in sympatry or in allopatry and migrated on multiple genetic backgrounds into sympatry after secondary contact, there may not be a strong signature of selection in sympatry or significant genetic divergence between allopatric and sympatric populations at these loci. However, increased genetic divergence between species in sympatry is likely to still occur regardless of the history of the mutations.

### 4.2. Recombination

The success of reinforcement (under the two-allele model) depends on high linkage disequilibrium between reinforcement alleles and alleles causing postzygotic reproductive isolation [97,101]. Alleles conferring increased prezygotic reproductive isolation are adaptive if they act to pair mutually compatible alleles. Recombination may cause these alleles to associate with opposing incompatibility alleles, resulting in incompatible pairings [32,101]. If recombination breaks down linkage disequilibrium between alleles causing prezygotic and postzygotic barriers then selection against hybrids can cause the local extinction of a species, or selection can eliminate hybrid incompatibilities and facilitate fusion of the sympatric populations [102,103]. Thus, reinforcement will be more likely to be successful if recombination is reduced between reinforcement genes and genes causing postzygotic reproductive isolation through tight physical linkage or their presence in regions of reduced recombination [101,104,105].

Recombination rates vary widely across the genome, both within and between chromosomes, as well as across species [36]. If a mutation causing increased prezygotic reproductive isolation arises in an area of low recombination it will be more likely to maintain linkage disequilibria with neighboring alleles causing species differences [106]. In this way, genomic regions of reduced recombination can harbor hybrid incompatibilities, species-specific adaptive alleles, and reinforcement alleles to allow persistence of linkage disequilibria in the face of gene flow. In particular, sex chromosomes and chromosomal rearrangements between species have reduced recombination rates [27,107], and therefore can facilitate the success of reinforcement alleles [108,109]. Empirical studies lend support to the importance of reduced recombination regions for the success of reinforcement. In two sympatric *Ficedula* flycatcher species, loci causing hybrid incompatibilities, male plumage color variation, and female species recognition all map to the Z chromosome [42]. Similarly, in two *Drosophila* species hybrid sterility, courtship displays, and female species preferences are all associated with inverted regions on one X chromosome and one autosome [108].

Genomic regions with reduced recombination tend to show patterns of elevated nucleotide diversity and sequence divergence across populations similar to those caused by strong divergent selection. Regions of low recombination rate experience increased genetic hitchhiking near selected loci; background selection can reduce nucleotide diversity within a population at these regions [36]. In addition, lower rates of gene exchange between populations due to reduced recombination cause higher sequence divergence due to drift. Therefore, genome scans for signatures of selection may mistake regions of low recombination for loci under strong divergent selection [71,110]. The effects of reduced recombination are expected to be weaker when gene flow is high; therefore, the signal of divergent selection in comparisons between allopatric and sympatric populations of the same species might be more evident than in comparisons between sympatric populations of different species.

To distinguish true signatures of selection for reinforcement from false positives due to background selection in areas of reduced recombination, we must consider the hallmarks of reinforcement: evidence of selective sweeps in sympatry that are absent in allopatry, and higher genetic divergence between sympatric and allopatric populations. Assuming genome–wide recombination rates are similar in allopatric and sympatric individuals of the same species, a signature of a selective sweep due to variation in recombination rates should occur in both allopatric and sympatric populations. Similarly, we do not necessarily expect higher divergence between allopatric and sympatric populations than between two sympatric populations in regions of low recombination. In fact, selective sweeps due to reinforcement in low recombination regions may be easier to detect because larger blocks of the genome will hitchhike to high frequency than they would in high recombination areas. However, isolating the causal mutations within these blocks may be more difficult [36].

The discussion above assumes that members of the same species do not differ substantially in their recombination rate. Polymorphic chromosomal rearrangements or variation in recombination rate between populations can confound this assumption [107]. For example, if sympatric and allopatric populations differ in the arrangement of an inversion, they may display high sequence divergence akin to a reinforcement scenario [111,112]. Also, a recently occurring inversion limited to the sympatric zone may exhibit reduced nucleotide diversity [113] matching our hypothetical reinforcement signature. While an inversion fitting this description may be a false positive, it is likely either a true carrier of reinforcement traits or is under divergent selection for another reason, such as local adaptation or ecological trait displacement. In fact, as stated above, if a reinforcement allele does get captured by an inversion along with alleles causing postzygotic reproductive isolation, this inversion will be favored in sympatry (although not in allopatry) due to reduced recombination between alleles causing reproductive isolation.

## 5. Gene Flow and the Evolution of Reinforcement

Genomic analyses of reinforcement can be used to identify causal genomic regions, but they can also inform the conditions under which reinforcement occurred. The evolution of reinforcement is rarely discussed without considering gene flow. The ability for new reproductive isolating barriers to evolve in sympatry is dependent on the extent of gene flow between sympatric species [7,114], thus gene flow can hinder or prevent the evolution of reinforcement [48,97]. With genomic analyses, we can discern if and how much gene flow occurs during reinforcement.

### 5.1. Gene Flow between Species

As discussed above, for new alleles causing reproductive isolation to evolve, they must maintain linkage disequilibrium with other species-specific alleles that cause local adaptation and incompatibilities [101,115]. Gene flow and recombination between sympatric species can disassociate alleles from their beneficial species-backgrounds and prevent the evolution of reproductive isolation. Extensive theory describes the parameter values (e.g., strong selection and weak gene flow) under which reinforcement can successfully occur [92,104,116,117]. However, empirical investigations of gene flow during reinforcement are rare.

With advancements in genomic analyses, we can now infer if and to what extent gene flow occurs or has occurred during reinforcement [96,118,119]. For example, there is genomic evidence of gene flow in the aforementioned example of reinforcement in *Phlox*. Patterns of genomic variation across species indicate gene flow between *Phlox* species in sympatry, but no evidence of gene flow with allopatric *P. drummondii* and *P. cuspidata* [120]. This geographic pattern of gene flow is consistent with reinforcement evolving in sympatry despite gene flow between *Phlox* species. This study did not have the resolution to estimate timing of gene flow, but promising genomic analyses suggest this type of inference could be possible in future studies of reinforcement [121].

**Figure 3:**
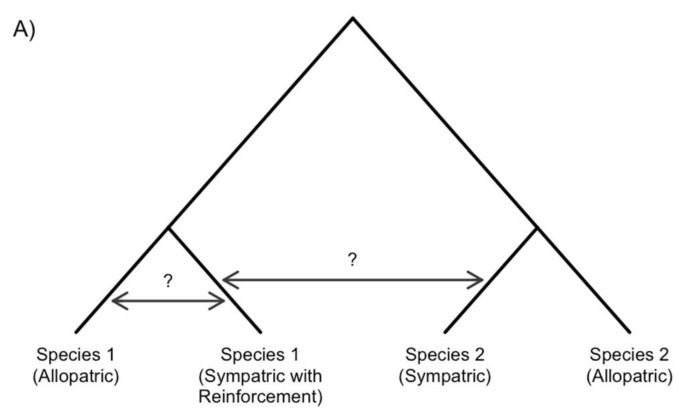

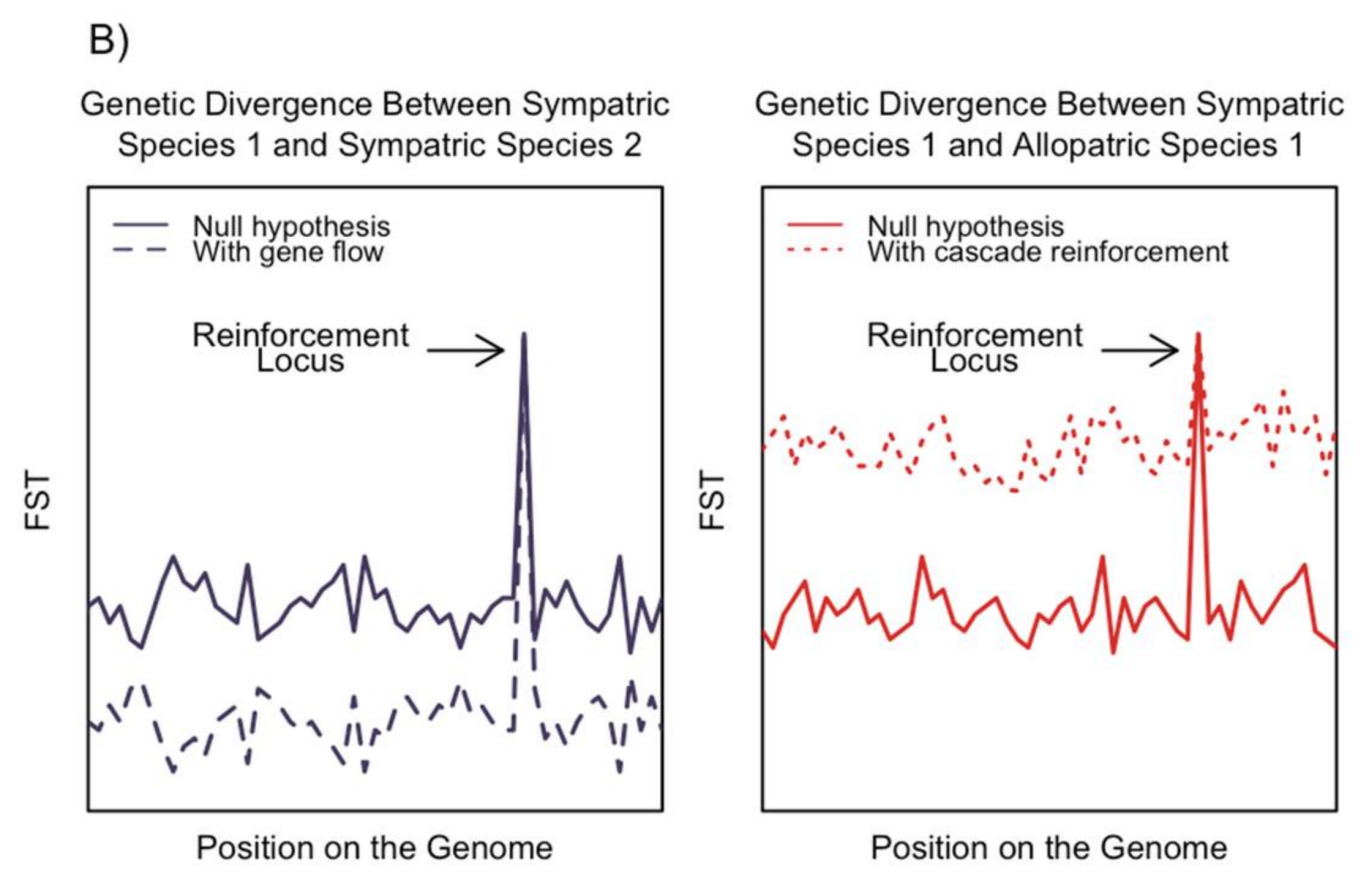
Gene flow affects the evolution of reinforcement (**a**) a phylogenetic network analysis of two species with sympatric and allopatric populations can allow for the identification of the direction and quantity of gene flow between the lineages. (**b**) Hypothetical examples of how gene flow, or the lack thereof, may impact genome-wide divergence between sympatric species. (**Left**) Gene flow between sympatric species (dashed) will decrease genome-wide genetic divergence, making identification of the reinforcement locus easier. (**Right**) Hypothetical example of how a lack of gene flow between allopatric and sympatric populations within a species impacts identification of reinforcement loci. Over time, cascade reinforcement (dotted) may increase the amount of genetic divergence observed between the allopatric and sympatric populations, making identification of the reinforcement locus more difficult.

A variety of statistical methods can be used to detect gene flow between populations and species [122,123,124,125]. For example, model-based methods such as STRUCTURE [126] and ADMIXTURE [127] deduce ancestry from population variation data. Summary statistics such as Patterson’s D [128] and F4 [129] use phylogenomic inference, and phylogenetic network methods, such as PhyloNetworks [130], test alternative models of reticulate evolution to determine the best fit given a distribution of gene trees (Figure 3a). Furthermore, demographic model choice methods can infer a history of gene flow, using likelihood [131], Approximate Bayesian Computation [132,133], or diffusion approaches [134]. Together these methods can provide novel insights into the timing, magnitude, and direction of gene flow. Having a detailed understanding of gene flow during reinforcement will significantly improve our understanding of how well the process empirically matches our theoretical expectations of how reinforcement evolves.

If gene flow does occur during the evolution of reinforcement, it will also have a pivotal role in shaping the patterns reinforcing selection leaves on the genome. Theory suggests that gene flow between species creates heterogeneous levels of genetic divergence across the genome. Loci under divergent selection (e.g., species-specific adaptation and hybrid incompatibilities) will have low effective migration and are likely to be highly differentiated, while neutral loci can recombine into either species’ genome without constraint [39,135]. Gene flow between sympatric species may therefore make loci causing reinforcement more prominent because the background levels of divergence between sympatric species will be lower (Figure 3b).

### 5.2. Gene Flow within Species

Gene flow is likely to occur between sympatric and allopatric populations within a species. If the trait causing increased reproductive isolation in sympatry is neutral, beneficial, or linked to an advantageous trait, the causal allele may spread into allopatry [7,116,136]. In this case, reinforcement will not cause the phenotypic or genomic patterns associated with character displacement. This situation may result in a signature of selection at the reinforcement locus in both sympatry and allopatry, low genetic divergence between conspecific populations, and similar genetic divergence between heterospecific sympatric and allopatric populations.

Trait evolution caused by reinforcement is often detrimental in allopatry, causing divergence between allopatric and sympatric populations. The divergence between allopatric and sympatric populations in mate preference or assortative mating traits can create reproductive isolation between conspecifics in divergent populations. Reduced gene flow between allopatric and sympatric populations due to this ‘cascade reinforcement’ could drive further genetic divergence that eventually leads to speciation [32,137]. Cascade reinforcement has been documented in some cases of reinforcement (e.g., [17,25,138,139,140,141,142,143]); however, its prevalence in nature remains unknown.

Cascade reinforcement will not interfere with a signature of selection in sympatry due to reinforcement, or genetic divergence between sympatric individuals of different taxa. However, over time, the reduced interbreeding between allopatric and sympatric populations of the same species due to cascade reinforcement will allow for accumulation of genetic divergence between the conspecific populations making the genetic differentiation at the reinforcement locus become less distinct (Figure 3b). If cascade reinforcement has occurred, identification of the original locus underlying reinforcement will be difficult without testing the function of all candidate regions.

Furthermore, sympatric and allopatric populations are, by definition, geographically isolated and thus migration and gene flow may be limited between these regions. Under this circumstance, the genome-wide divergence between sympatric and allopatric populations could increase over time and mirror the effects of cascade reinforcement.

## 6. Conclusions

Advancements in next-generation sequencing technology and genomic analyses have revolutionized our understanding of the genetic basis of reproductive isolation and the process by which speciation occurs [38]. However, reinforcement has rarely been studied at the genomic level. Here we describe three patterns of genomic variation that may result from reinforcement. Genome scans for these patterns may help to identify if reinforcement has occurred, as well as provide insights into the genetic basis of previously identified cases of reinforcement. Detailed analyses of genetic variation can yield insight into the nature and origin of a reinforcement allele, if and to what extent reinforcement evolved with gene flow, and how the genomic architecture has facilitated its evolution.

As with any genomic analysis, there are many caveats to interpreting genomic patterns consistent with reinforcement. There are other evolutionary processes and characteristics of genome structure that can create similar patterns of genomic variation. Genomic analyses can complement but not replace ecological studies that measure selection and reproductive isolation, and molecular studies that validate the function of genomic variation. Nonetheless, with new molecular tools, sequencing technologies, and genomic analyses, genomic studies of reinforcement can significantly improve our understanding of how reinforcement occurs during speciation.
